# Transcriptional co-activator TAZ sustains proliferation and tumorigenicity of neuroblastoma by targeting CTGF and PDGF-β

**DOI:** 10.18632/oncotarget.3367

**Published:** 2015-03-24

**Authors:** Mei Wang, Yang Liu, Jiahua Zou, Rui Yang, Fan Xuan, Yi Wang, Ning Gao, Hongjuan Cui

**Affiliations:** ^1^ State Key Laboratory of Silkworm Genome Biology, Southwest University, Chongqing, China; ^2^ Department of Respiration, the Third Hospital of Hebei Medical University, Shijiazhuang, China; ^3^ Cardiovascular Department, Second Affiliated Hospital of University of South China, Hengyang, China; ^4^ Department of Pharmacognosy, College of Pharmacy, Third Military Medical University, Chongqing, China

**Keywords:** TAZ, cell proliferation, colony formation, neuroblastoma

## Abstract

Neuroblastoma is a common childhood malignant tumor originated from the neural crest-derived sympathetic nervous system. A crucial event in the pathogenesis of neuroblastoma is to promote proliferation of neuroblasts, which is closely related to poor survival. However, mechanisms for regulation of cell proliferation and tumorigenicity in neuroblastoma are not well understood. Here, we report that overexpression of TAZ in neuroblastoma BE(2)-C cells causes increases in cell proliferation, self renewal and colony formation, which was restored back to its original levels by knockdown of TAZ in TAZ-overexpression cells. Inhibition of endogenous TAZ attenuated cell proliferation, colony formation and tumor development in neuroblastoma SK-N-AS cell, which could be rescued by re-introduction of TAZ into TAZ-knockdown cells. In addition, we found that overexpressing TAZ-mediated induction of CTGF and PDGF-β expression, cell proliferation and colony formation were inhibited by knocking down CTGF and PDGF-β with siRNA in TAZ-overexpressing cell. Overall, our findings suggested that TAZ plays an essential role in regulating cell proliferation and tumorigenesis in neuroblastoma cells. Thus, TAZ seems to be a novel and promising target for the treatment of neuroblastoma.

## INTRODUCTION

Neuroblastoma is a common malignant tumor of a developing sympathetic nervous system associated with a poor prognosis, resulting in approximately 15% of childhood cancer-related deaths [1–6]. Despite surgical, radio and chemo-therapy have improved the outcomes of neuroblastoma patients, nearly 50% of children with high-risk neuroblastoma have a relapse, and to date there are no curative salvage treatment regimens [4]. Over the past decades, although many tumor suppressor genes (e.g. *p53*, *Rb* and *p21*) and oncogenes (e.g. *MYCN*, *c-sis*) have been identified to be important for the development of neuroblastoma [7–10], little was known about the genetic basis of neuroblastoma. Therefore, the identification of new proteins responsible for the development of neuroblastoma is critical for the development of novel therapeutic strategies for treating neuroblastoma.

TAZ is a WW-domain-containing transcriptional co-activator, which is important for development of various tissues in mammals [11, 12]. TAZ has also been shown to bind with a variety of transcription factors such as the RUNX family, TEAD, TTF-1, TBX5 and Pax3 [13–16]. Recently, TAZ was identified as a component of the emerging Hippo-LATS tumor suppressor pathway, which is involved in regulating the transcriptional outcome to govern cell proliferation and apoptosis [17]. Most recently, enhanced expression of TAZ has been found in both breast cancer and non-small cell lung cancer (NSCLC) cells [18, 19]. In addition, overexpression of TAZ has been shown to induce cell proliferation and tumorigenesis in breast cancer and NSCLC cells, whereas knocking down TAZ expression in breast cancer and NSCLC cells suppresses cell proliferation and tumorigenesis, suggesting that TAZ may function as an oncogene in breast cancer and NSCLC. However, the role of TAZ in regulating cell proliferation and tumorigenesis in neuroblastoma cells has not been explored.

In this study, we provided the evidence that overexpression of TAZ induced cell proliferation and tumorigenicity in neuroblastoma, whereas knockdown of TAZ inhibited cell proliferation and tumorigenicity in neuroblastoma. Mechanistically, we found that TAZ promotes cell proliferation and tumorigenicity through up-regulating the expression of CTGF and PDGF-β genes. Our findings demonstrate that TAZ may act as a critical regulator of neuroblastoma cell proliferation and tumorigenicity, and seems to be a novel and promising target for the treatment of neuroblastoma.

## RESULTS

### High TAZ expression is prognostic of poor survival in neuroblastoma patients

**Figure 1:**
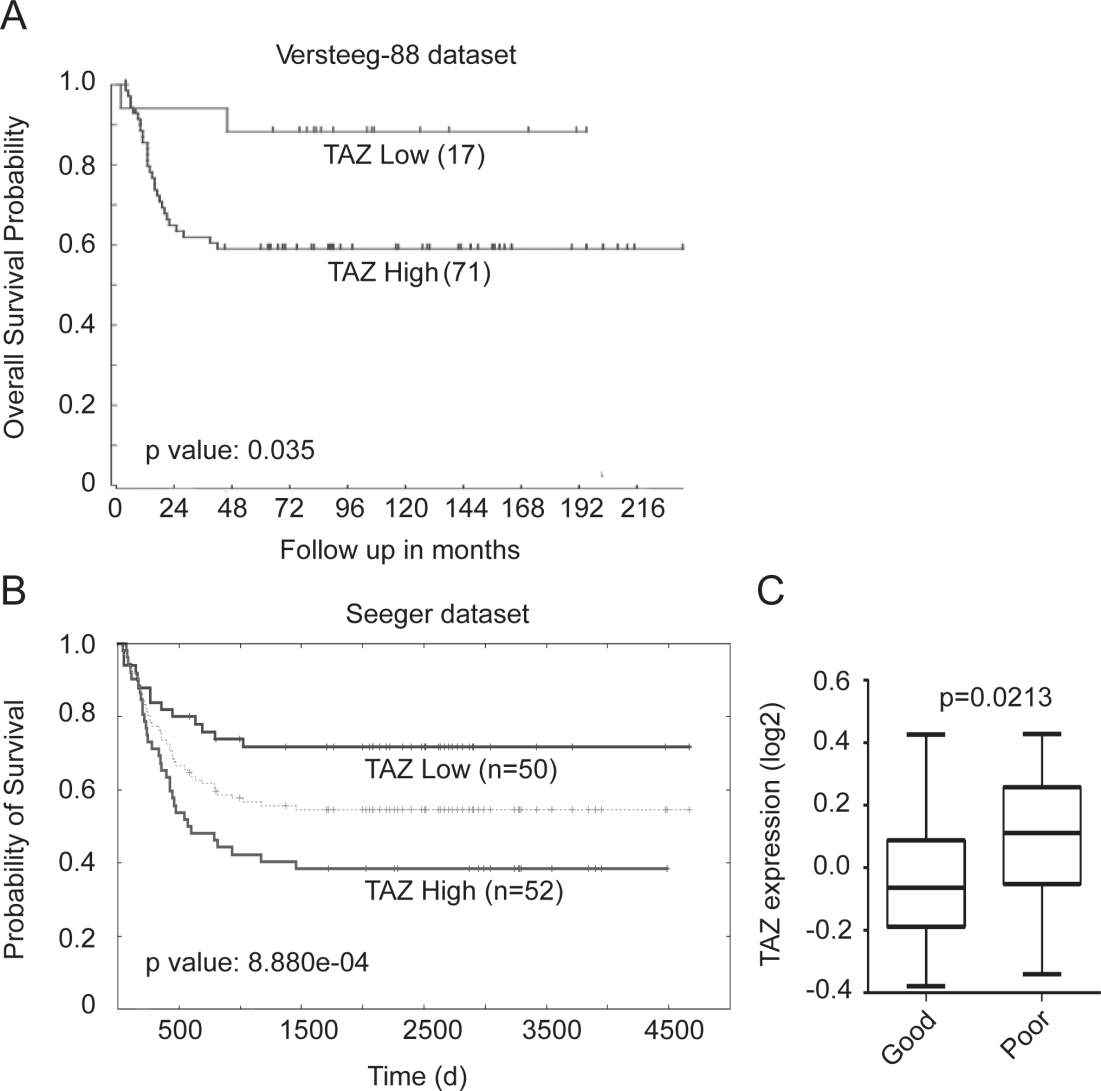
TAZ expression is prognostic of worse survival in neuroblastoma patients **(A)** Kaplan-Meier analysis of progression-free survival for Versteeg dataset with log-rank test *P*-values indicated. The TAZ expression cutoff value 0.035 was determined by the online R2: microarray analysis and visualization platform, which separated patients into high and low TAZ expression groups. **(B)** Kaplan-Meier analysis of the Seeger dataset with the log-rank test *P* value indicated. **(C)** Box plot of TAZ expression levels in tumor good and poor prognosis groups.

To investigate the prognostic value of TAZ in neuroblastoma, the Tumor Neuroblastoma public-Versteeg database, which is available from the online R2: microarray analysis and visualization platform were performed. The Versteeg database contains a cohort of 88 patients with neuroblastoma representative of survival prognosis. 71 patients with high TAZ expression showed low survival probability, whereas 17 patients with low TAZ expression showed high survival probability (Figure 1A), suggesting that high TAZ expression is prognostic for poor outcome of neuroblastoma patients. Kaplan-Meier analysis of progression-free survival for the Seeger dataset also confirmed that high TAZ expression was associated with poor outcome, whereas low TAZ expression was associated with good prognosis (Figure 1B and 1C). Together, our analysis of two independent datasets indicated that TAZ could be a potential prognostic marker in neuroblastoma.

### Expression of TAZ in neuroblastoma cell lines

**Figure 2:**
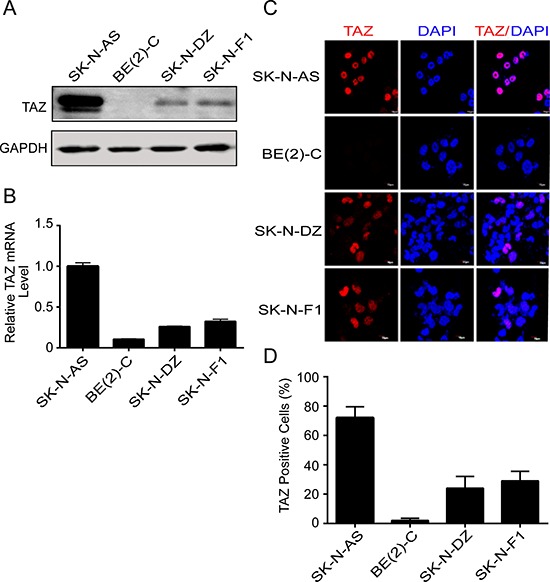
TAZ is commonly expressed in neuroblastomas **(A, B)** Four neuroblastoma cell lines SK-N-AS, BE(2)-C, SK-N-DZ, and SK-N-F1 were harvested and subjected to Western blot and qRT-PCR to detect TAZ expression. **(C)** Four neuroblastoma cell lines were fixed and immunostained with TAZ monoclonal antibody (red), nuclei were counterstained with DAPI (blue), and evaluated by immunofluorescent microscopy. **(D)** Quantitative analysis was performed to evaluate the percentage of TAZ-positive cells in four neuroblastoma cells. Data are presented as mean ± S.D. from three independent experiments.

Since the elevated expression of TAZ has been found in various cancers [20, 21], we next examined TAZ expression in four neuroblastoma cell lines including SK-N-AS, BE(2)-C, SK-N-DZ and SK-N-F1 cells using Western blot and real-time RT-PCR. As shown in Figure 2A and 2B, TAZ was expressed at varying levels of protein and mRNA in all neuroblastoma cell lines. Among the neuroblastoma cell lines, high level of TAZ was detected in SK-N-AS cells, whereas moderate levels of TAZ were observed in SK-N-DZ and SK-N-F1 cells and low level of TAZ was found in BE(2)-C cells. Consistent with these results, immunofluorescent labeling also revealed that the expression of TAZ was found in neuroblastoma cells (Figure 2C). Quantitative analysis indicated that the percentage of TAZ-positive cells was accounted for 72%, 2%, 24% and 29% in SK-N-AS, BE(2)-C, SK-N-DZ and SK-N-F1 cell lines, respectively (Figure 2D). These observation demonstrated that TAZ is indeed expressed in neuroblastoma cells.

### Overexpression of TAZ increases cell proliferation and colony formation

**Figure 3:**
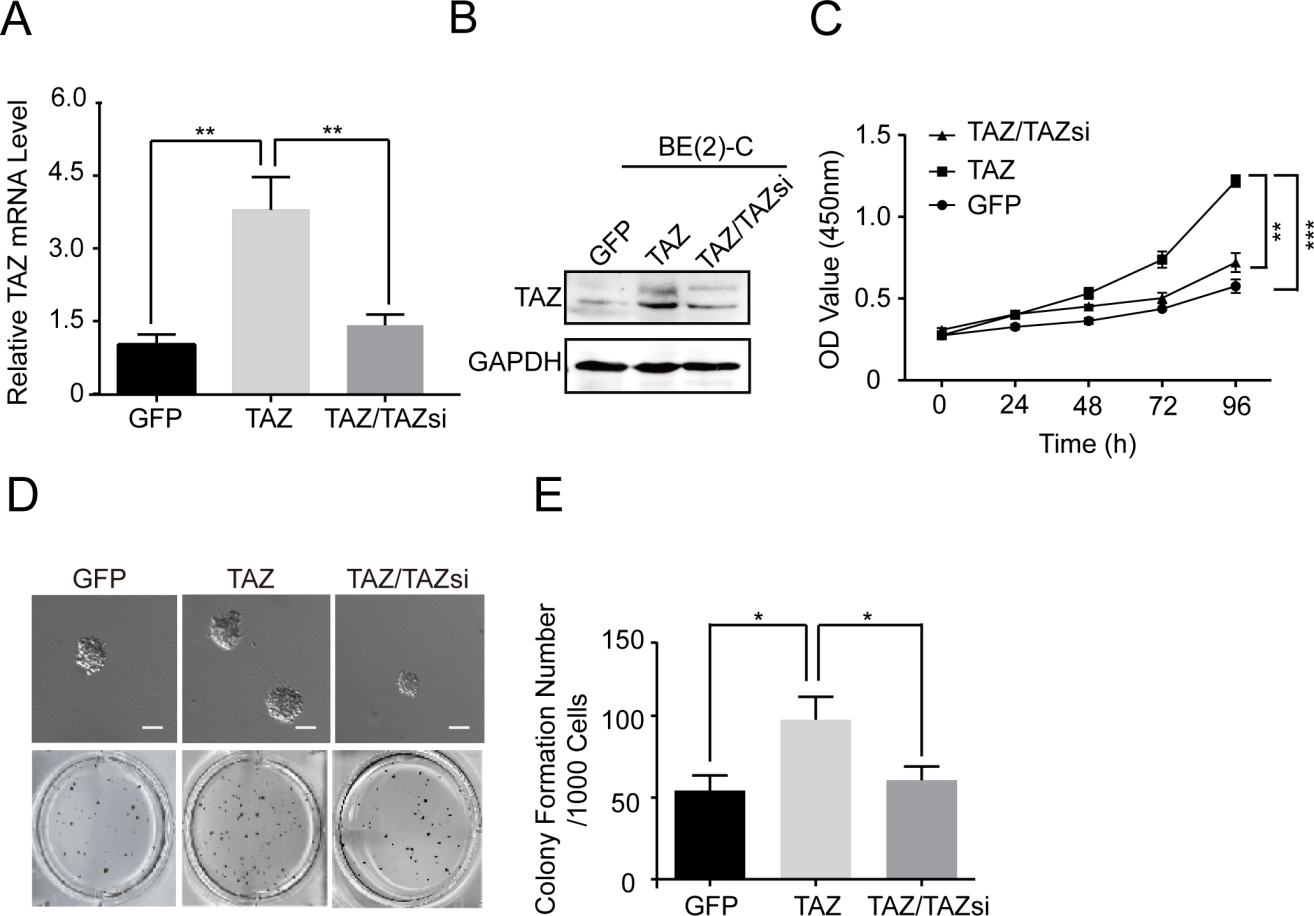
Overexpression of TAZ promotes cell proliferation and colony formation BE(2)-C cells were transfected with vector control (GFP), TAZ and TAZ/TAZsi. **(A)** Total RNA was isolated and TAZ mRNA was quantified using qRT-PCR analysis. The values represent the means ± SD from three separate experiments. ** Values for cells transfected with TAZ are significantly increased compared with those for control cells or cells double transfected with TAZ and TAZ siRNA by the Student's *t* test; *P* < 0.01. **(B)** Total cellular extracts were prepared and subjected to Western blot using antibody against TAZ. **(C)** BE(2)-C cells were seed into a 96-well plate (1000 cells/well), and cell proliferation was determined using cell counting kit-8 assay kit. Data represent the means ± SD from three independent experiments (**P < 0.01, ***P < 0.001). **(D)** 1000 cells were mixed with 0.6% agar and 2 fold DMEM medium, and overlaid on 1.2% agar mixed with 2 fold DMEM medium in a 34.8 mm plate. Colony formation was examined by staining colonies with 200 μl MTT per well. **(E)** Colony number was counted using counter. The values represent the mean ± SD from three independent experiments. * Values for TAZ-overexpressed BE(2)-C cells are significantly higher than those expressing GFP vector or TAZ/TAZsi by the Student's *t* test; *P* < 0.05.

To explore the function of TAZ in neuroblastoma, we investigated the effects of overexpressing TAZ on cell proliferation and colony formation in a TAZ-low expression cell line (BE(2)-C). We overexpressed TAZ by lentivirus-mediated infection of BE(2)-C cells with vector alone or TAZ. Western blot and real time RT-PCR showed that the protein and mRNA levels of TAZ in TAZ-overexpressing cells are more than 3-fold of those in vector control cells (Figure 3A and 3B). Overexpression of TAZ in BE(2)-C cells significantly enhanced cell proliferation compared with vector control cells (Figure 3C). Furthermore, overexpression of TAZ markedly increased the growth of BE(2)-C cell on soft agar (Figure 3D and 3E).

To eliminate the possibility that enhanced cell proliferation and colony formation of BE(2)-C cells are caused by nonspecific effect of the lentivirus rather than TAZ overexpression, we knocked down TAZ in BE(2)-C-TAZ cells back to its original levels by short-hairpin RNA (shRNA) against TAZ (Figure 3A and 3B). Significantly, knocking down TAZ reversed TAZ-induced cell proliferation and colony formation (Figure 3C and 3D). Taken together, these results suggest that TAZ plays a critical role in regulating cell proliferation and tumorigenesis in neuroblastoma cells.

### Knocking down TAZ in SK-N-AS cells suppresses cell proliferation and colony formation

**Figure 4:**
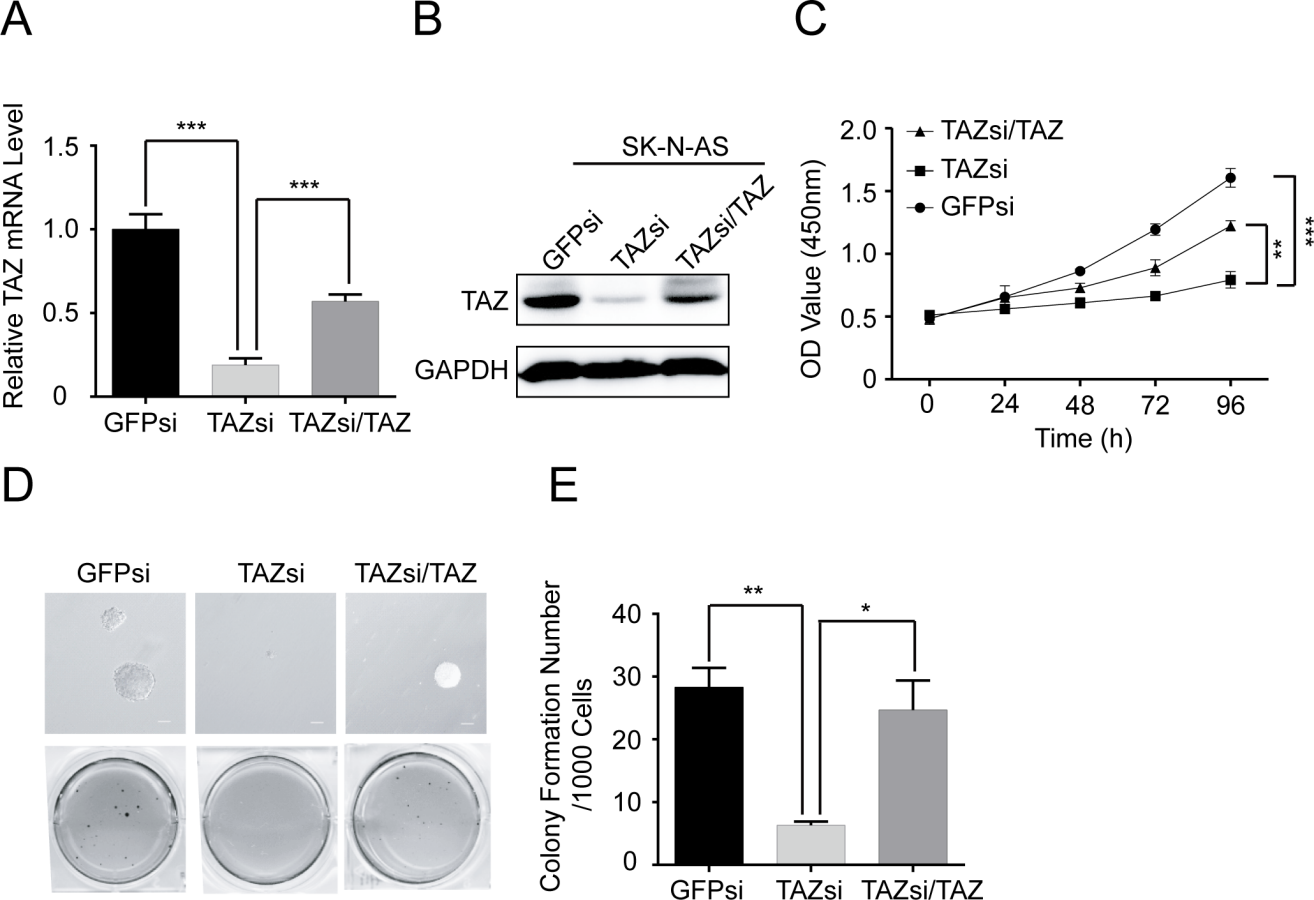
Knockdown of TAZ inhibits cell proliferation and colony formation SK-N-AS cells were transfected with control siRNA, TAZ siRNA or TAZ siRNA/TAZ. **(A)** Total RNA was isolated and TAZ mRNA was quantified using qRT-PCR analysis. Data represent the means ± SD from three independent experiments (**P < 0.001). **(B)** Total celluler extracts were prepared and subjected to Western blot using antibody against TAZ. **(C)** SK-N-AS cells were seed into a 96-well plate (1000 cells/well), and cell proliferation was determined using cell counting kit-8 assay kit. Data represent the means ± SD from three independent experiments (**P < 0.01; ***P < 0.001). **(D)** Colony formation was examined by soft agar assay. **(E)** Colony number was counted using counter. The values represent the mean ± SD from three independent experiments (*P < 0.05 or **P < 0.01).

To determine whether elevated levels of TAZ directly contribute to tumorigenicity of neuroblastoma cells, we knocked down TAZ in SK-N-AS cells, which exhibit high endogenous levels of TAZ, by infecting with lentivirus-expressing shRNA targeting vector control (pLKO.1) or TAZ. Knockdown of endogenous TAZ was confirmed by Western blot and qRT-PCR analysis. As shown in Figure 4A and 4B, the protein and mRNA levels of TAZ were significantly decreased in SK-N-AS cells infected with TAZ shRNA compared with those in control shRNA cells. Knockdown of TAZ in SK-N-AS cells markedly suppressed cell proliferation (Figure 4C). In addition, knockdown of TAZ in SK-N-AS caused significant decreases in anchorage-independent growth on soft agar (Figure 4D).

To further confirm that the effect of TAZ knockdown on cell proliferation and colony formation is not an off-target or viral effect, we next overexpressed TAZ in TAZ-knocked down SK-N-AS cells, in which the protein and mRNA levels of TAZ were back to those in SK-N-AS cells (Figure 4A and 4B). Overexpression of TAZ restored cell proliferation and colony formation in the TAZ knockdown cells (Figure 4C–4E), suggesting that the reduced cell proliferation and colony formation mediated by knocking down TAZ are due to the reduction of TAZ in neuroblastoma cells. These findings further confirm the important role of TAZ in regulating cell proliferation and tumorigenesis in neuroblastoma cells.

### Knocking down TAZ suppresses tumor growth of neuroblastoma *in vivo*

**Figure 5:**
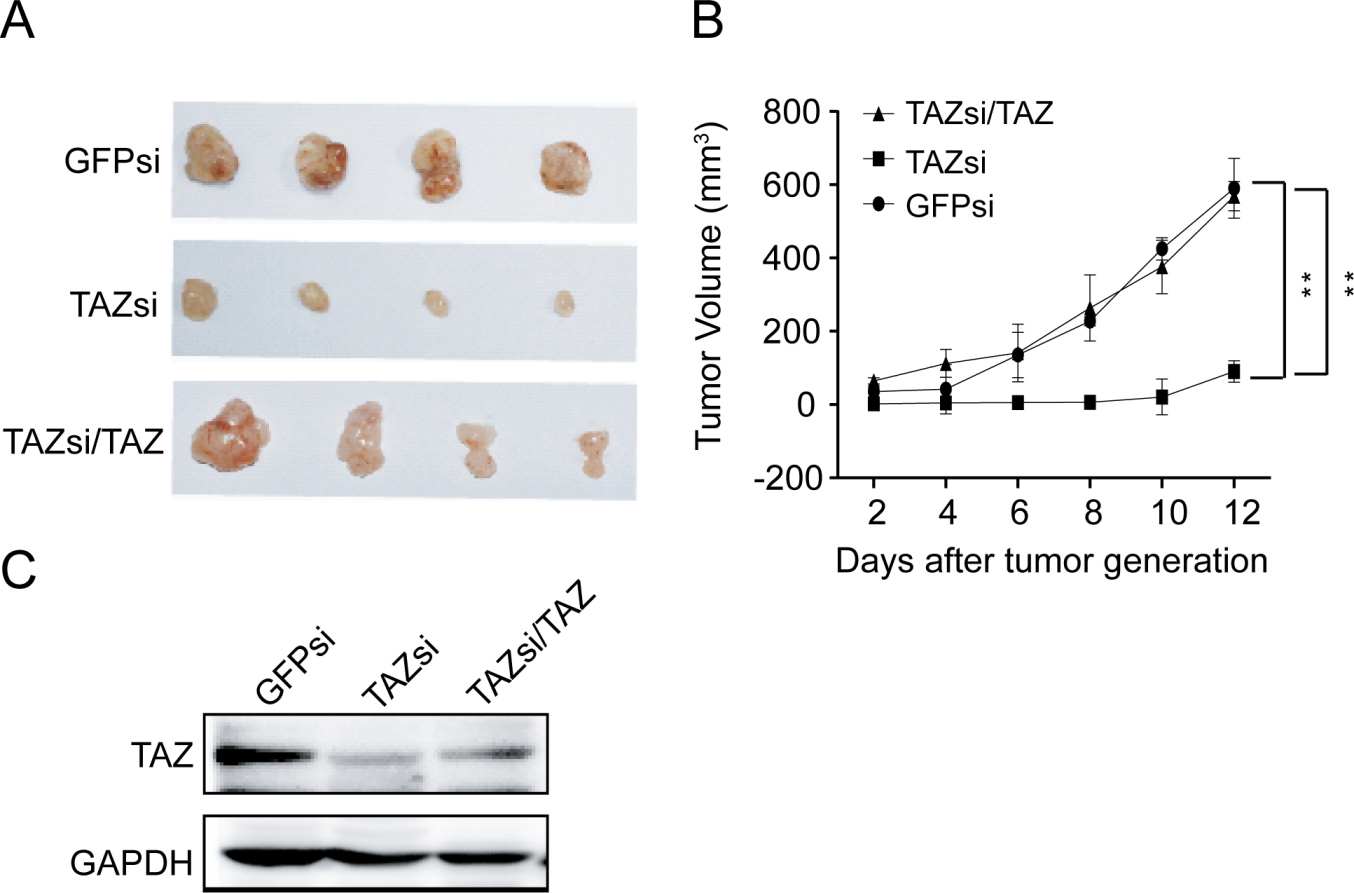
TAZ is essential for neuroblastoma tumor growth *in vivo* **(A)** The NOD/SCID mice xenograft tumor inoculated with GFPsi, TAZsi and TAZsi/TAZ cells. **(B)** Average tumor volume in mice injected with control GFPsi, TAZsi and TAZsi/TAZ cells. Error bars represent means ± SD. **P < 0.01, TAZsi group compared with control GFPsi or TAZsi/TAZ. **(C)** Western blot analysis was performed to determine TAZ expression in tumor tissues of mouse neuroblastoma xenografts.

To investigate whether TAZ is also important for the tumorigenicity of neuroblastoma cell *in vivo*, NOD/SCID mice were subcutaneously inoculated with SK-N-AS cells expressing vector control siRNA (GFPsi) or TAZ siRNA. Significantly, knocking down TAZ in SK-N-AS cells dramatically suppressed tumor growth of neuroblastoma xenograft in NOD/SCID mice (Figure 5A and 5B). To further confirm that the effect of knockdown of TAZ on tumorigenicity is not an off-target or viral effect, we next overexpressed TAZ in TAZ-knocked down SK-N-AS cells and subcutaneously inoculated these cells into NOD/SCID mice. Overexpression of TAZ restored the tumorigenicity inhibited by knocking down TAZ (Figure 5A and 5B), suggesting that enhanced levels of TAZ may be responsible for the tumorigenesis of neuroblastoma cells.

To further confirm the critical role of TAZ in the tumorigenesis of neuroblastoma cells, Western blot was employed. Decreased levels of TAZ was observed in tumor of NOD/SCID mice inoculated with TAZ siRNA cells compared with that in NOD/SCID mice inoculated with control siRNA cells (Figure 5C). Collectively, these findings indicate that TAZ enhanced the tumorigenesis of neuroblastoma *in vivo*.

### CTGF and PDGF-β are the major downstream transcriptional targets of TAZ

**Figure 6:**
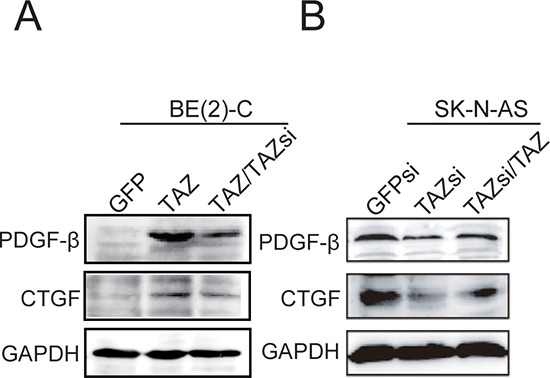
Activation of CTGF and PDGF-β by TAZ **(A)** BE(2)-C cells were overexpressed with TAZ or overexpressed TAZ cells were knocked down with TAZ siRNA. Total cellular extracts were prepared and subjected to Western blot using antibodies against CTGF and PDGF-β. **(B)** SK-N-AS cells were knocked down with TAZ siRNA or TAZ siRNA cells were re-introduced with TAZ. Total cellular extracts were prepared and subjected to Western blot using antibodies against CTGF and PDGF-β. GAPDH levels are shown as loading control.

To explore the molecular mechanism underlying TAZ-induced cell proliferation and tumorigenesis, we identified the downstream targets of TAZ. Connective tissue growth factor (CTGF, also known as CCN2) is a secreted protein that acts as ligands of integrins to regulate cell proliferation, migration, apoptosis, and angiogenesis [22]. Platelet-derived growth factor-β (PDGF-β) is a *c-sis* proto-oncogene, which is important for embryonic development, cell proliferation and differentiation [23, 24]. Recently, CTGF has been identified as the transcriptional target of TAZ [25, 26]. However, PDGF-β has not been identified as a transcriptional target of TAZ. The functional significance of the regulation of CTGF and PDGF-β by TAZ in neuroblastoma cells is unknown. To confirm whether both CTGF and PDGF-β are certainly downstream targets of TAZ, we determined the expression of CTGF and PDGF-β in TAZ overexpressing BE(2)-C cells using Western blot analysis. The elevated levels of CTGF and PDGF-β were observed in TAZ overexpressing BE(2)-C cells (Figure 6A). Most interestingly, enhanced CTGF and PDGF-β protein were reduced back to their original levels when overexpressed TAZ in BE(2)-C cells was knocked down by TAZ siRNA (Figure 6A). We also determined whether knockdown of TAZ reduces the expression of CTGF and PDGF-β. Contrary to TAZ overexpression, knockdown of TAZ by siRNA (TAZsi) markedly decreased the expression of both CTGF and PDGF-β in SK-N-AS cells (Figure 6B). Moreover, decreased CTGF and PDGF-β protein were restored back to their original levels when knocked down TAZ in SK-N-AS cells was overexpressed with TAZ (Figure 6B).

**Figure 7:**
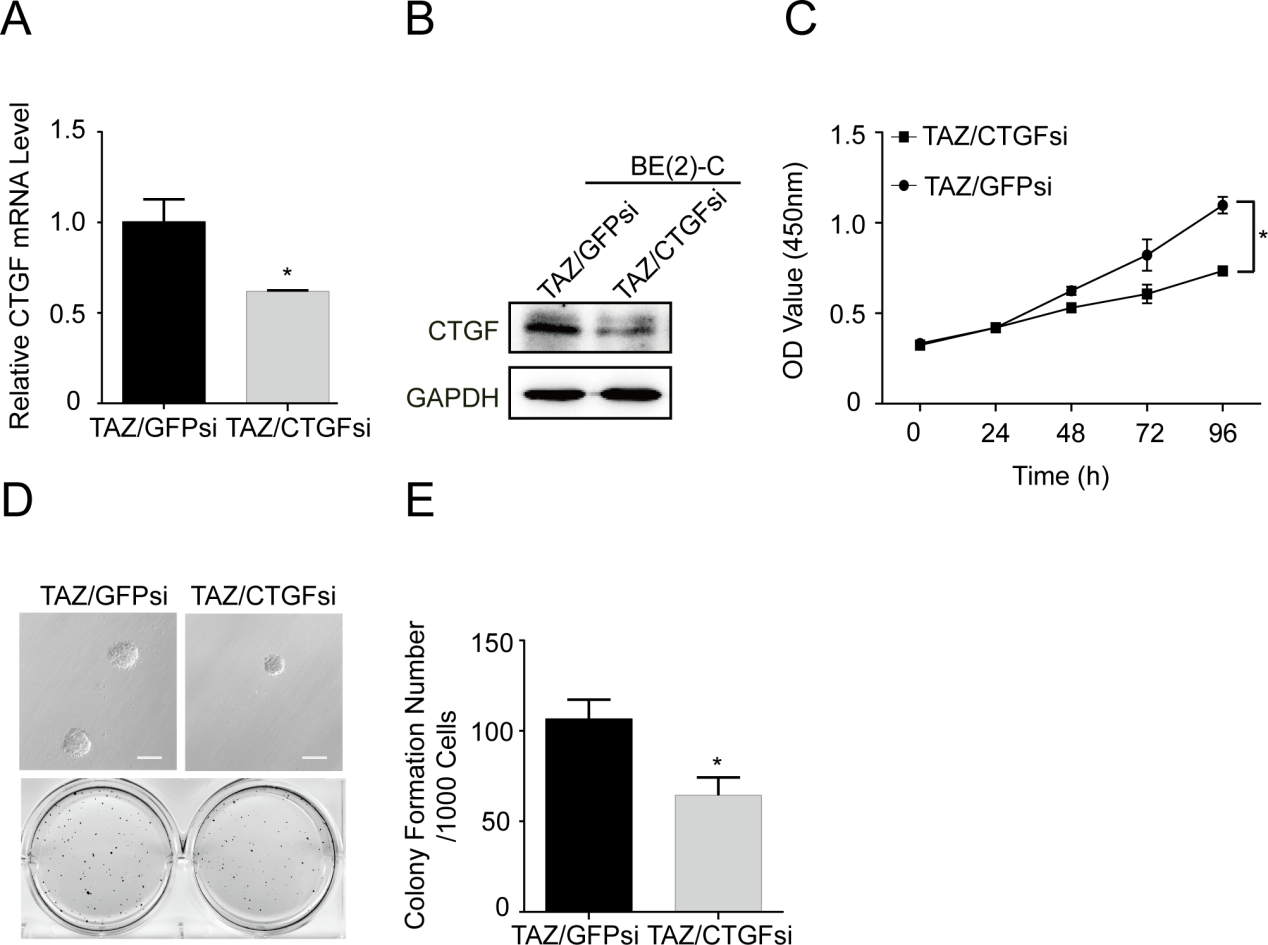
CTGF is a major downstream transcriptional target of TAZ TAZ overexpressing BE(2)-C cells were infected with CTGF siRNA. **(A)** qRT-PCR analysis was performed to determine the expression of CTGF at mRNA levels. Data represent the means ± SD from three independent experiments, *P < 0.05. **(B)** Western blot analysis was performed to determine the expression of CTGF. GAPDH levels are shown as loading control. **(C)** Cells were seed into a 96-well plate (1000 cells/well), after which cell proliferation was determined using cell counting kit-8 assay kit. Data represent the means ± SD from three independent experiments (*P < 0.05). **(D)** Colony formation was examined by soft agar assay. **(E)** Colony number was counted using counter. The values represent the mean ± SD from three independent experiments (*P < 0.05).

To examine the role of CTGF and PDGF-β in TAZ-induced proliferation and tumorigenesis, we respectively knocked down CTGF and PDGF-β in TAZ overexpressing BE(2)-C cells. Western blot and real time RT-PCR analysis showed that knocking down CTGF in TAZ overexpressing cells with CTGF siRNA decreased the protein and mRNA levels of CTGF in these cells (Figure 7A and 7B). Proliferation of TAZ overexpressing cell was suppressed when CTGF was knocked down by siRNA (Figure 7C). Knockdown of CTGF by siRNA (CTGFsi) in TAZ overexpressing cells also decreased the colony formation compared with vector control cells (Figure 7D and 7E).

**Figure 8:**
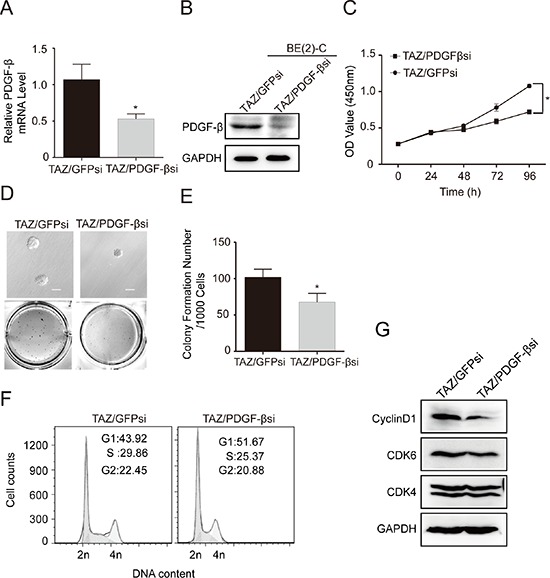
PDGF-β is a major downstream transcriptional target of TAZ TAZ overexpressing BE(2)-C cells were infected with PDGF-β siRNA. **(A)** qRT-PCR analysis was performed to determine mRNA expression of PDGF-β. Data represent the means ± SD from three independent experiments. (*P < 0.05). **(B)** Western blot analysis was performed to determine PDGF-β expression. GAPDH levels are shown as loading control. **(C)** Cells were seed into a 96-well plate (1000 cells/well), and cell proliferation was determined using cell counting kit-8 assay kit. Data represent the means ± SD from three independent experiments. (*P < 0.05). **(D)** Colony formation was examined by soft agar assay. **(E)** Colony number was counted using counter. The values represent the mean ± SD from three independent experiments (*P < 0.05). **(F)** Cell cycle was analyzed using PI staining and flow cytometry. **(G)** Immunoblot analysis was performed to determine the expression of G1 cell cycle regulatory proteins including cyclin D1, CDK4 and CDK6. GAPDH levels are shown as loading control.

Furthermore, we examined the effects of knocking down PDGF-β on cell proliferation and colony formation in TAZ overexpressing BE(2)-C cells by PDGF-β siRNA (Figure 8A and 8B). Knockdown of PDGF-β in TAZ overexpressing cells by PDGF-β siRNA markedly suppressed the cell proliferation (Figure 8C). In addition, knocking down PDGF-β in TAZ overexpressing cells partially caused decreases in anchorage-independent growth on soft agar (Figure 8D and 8E). Taking together, these findings strongly suggest that CTGF and PDGF-β are the downstream transcriptional factors of TAZ. To elucidate the mechanisms involved in TAZ-mediated proliferation of neuroblastoma cells, we examined the effects of knocking down PDGF-β on cell cycle in TAZ overexpressing BE(2)-C cells. Knockdown of PDGF-β in TAZ overexpressing cells partially inhibited cell proliferation by causing cell cycle arrest at G1 phase (Figure 8F). To gain insight into the molecular mechanism underlying downregulation of PDGF-β in inducing cell cycle arrest at G1 phase, we investigated the effects of knocking down PDGF-β on the expression of G1 cell cycle regulatory proteins. Knockdown of PDGF-β in TAZ-overexpressed cells led to decreases in levels of Cyclin D1 and CDK6, whereas the levels of CDK4 remained relatively unchanged (Figure 8G). Taken together, these findings suggest that TAZ promotes cell proliferation and tumorigenesis by transcriptional regulation of CTGF and PDGF-β.

## DISCUSSION

This study provides several lines of evidence that TAZ promotes cell proliferation and tumorigenesis in neuroblastoma cells. Firstly, the elevated protein and mRNA levels of TAZ were observed in neuroblastomas cells. Secondly, overexpression of TAZ in BE(2)-C cells increased cell proliferation and colony formation, whereas knocking down TAZ decreased cell proliferation and colony formation. On the other hand, knockdown of endogenous TAZ in SK-N-AS cells suppressed cell proliferation and colony formation, and overexpression of TAZ restored cell proliferation and colony formation in the TAZ knockdown cells. Lastly, knockdown of endogenous TAZ in SK-N-AS cells suppressed the tumor growth of neuroblastoma cell xenograft model. Our studies provided the first *in vitro* and *in vivo* evidence that TAZ is a novel oncogene and may have important roles in the development of neuroblastoma. Based on two independent gene expression datasets of neuroblastoma patients, we found that high TAZ expression is predicts for poor outcomes of neuroblastoma patients. Future development of reagents targeting TAZ will be promising therapeutic strategies for the successful treatment of neuroblastoma.

Although TAZ has been shown to have a role in regulating various biological functions, the molecular mechanism governing the function of TAZ remain largely unknown. It has recently been reported that several direct target genes of TAZ including *CTGF* and *Cyr61* have the important roles in TAZ-induced cell proliferation and tumorigenesis in mammary epithelial cells [26, 27]. Other TAZ downstream target genes including *IRS-1*, *MYLK*/*MLCK*, and *PLK2* are also important for tumor development and metastasis [28, 29]. In this study, we found that CTGF and PDGF-β are two major downstream transcriptional targets and have the critical roles in regulating cell proliferation and tumorigenesis based on the following evidence: Firstly, overexpression of TAZ in BE(2)-C cells increased protein levels of CTGF and PDGF-β. When overexpressed TAZ in BE(2)-C cells was knocked down by TAZ siRNA, enhanced CTGF and PDGF-β protein were reduced back to their original levels. Secondly, knockdown of TAZ in SK-N-AS cells decreased protein levels of CTGF and PDGF-β. When knocked down TAZ in SK-N-AS cells was overexpressed with TAZ, the decreased CTGF and PDGF-β protein levels were restored back to their original levels. Thirdly, knockdown of CTGF and PDGF-β blocked TAZ-induced cell proliferation and tumorigenesis in TAZ overexpressing neuroblastoma cells. Together, our studies provided the first biological evidence that overexpression of TAZ oncogene can induce cell proliferation and tumorigenesis through activation of two downstream transcriptional factors CTGF and PDGF-β.

TAZ is a transcriptional factor with various biological functions. TAZ itself has no DNA binding domain, therefore, it must bind to DNA binding transcriptional factors to stimulate downstream target gene expression. TAZ has been reported to bind many factors such as RUNX family, TTF-1, TBX5, PAX3, PAX8, peroxisome proliferator-activated receptor γ and TEAD [26, 30, 31]. Recently, it has been shown that the TEAD family is a major TAZ interacting transcriptional factor mediating cell proliferation, migration, and EMT induction [26, 32]. Interaction of TAZ with the TEAD family of transcriptional factors was essential for TAZ to promote transcription of the downstream genes *Cyr61*/*CTGF*, leading to Taxol resistance in breast cancer cells [33]. Among the TEAD family of transcriptional factors, TEAD4 is the most important TEAD in TAZ-mediated cell proliferation and tumorigenesis [26, 34]. TAZ promotes cancer cells proliferation, invasion, metastasis and chemo-resistance [31, 33, 34, 39, 40], and high expressions of TAZ has been observed in various human cancers such as breast cancer, lung cancer, colon cancer, liver cancer, pancreatic cancer, and is closely associated with poor prognosis [17, 37, 38]. In addition, TAZ not only regulates normal stem cell differentiation and self-renewal [41, 42], but also confers stem-cell-like traits to non-cancer stem cells (CSC). Nonetheless, silencing of TAZ in CSCs decreased the self-renewal capacity and tumor formation [19]. These findings hint that inhibiting TAZ and its related pathways may provide a promising strategy for the treatment of cancers.

In this study, our results showed that CTGF protein level was decreased in TAZ knockingdown cells, and the CTGF expression level was restored after TAZ was reintroduced in TAZ knockingdown cells. CTGF is a member of the connective tissue factor CCN (CTGF, Cyr61, Nov) family, which is a cysteine-rich, associated with extracellular matrix protein [43]. CCN family proteins share uniform modular structure which mediate various cellular functions such as regulation of cell division, chemotaxis, apoptosis, adhesion, motility, angiogenesis, neoplastic transformation, and ion transport. There is growing body of evidence that CTGF regulates cancer cell migration, invasion, angiogenesis, and apoptosis, which are closely associated with tumor development and progression [43–46]. Similar to TAZ, CTGF has previously been identified as an oncoprotein in glioma and breast cancer [43, 47]. Recently, CTGF has been identified as a direct target of TAZ [39]. Our results suggested that CTGF is a major downstream transcriptional target of TAZ mediating TAZ-induced cell growth and tumorigenicity in neuroblastoma cells.

Our results also showed that platelet-derived growth factor beta (PDGF-β) is another major transcriptional target of TAZ. PDGF-β was firstly isolated as a protein produced by platelets, and it stimulate DNA synthesis and growth of cells in culture [48]. The major function of PDGF-β is to promote and regulate cell proliferation, differentiation and migration, and is involved in both developmental processes and in maintaining tissue homeostasis [49, 50]. Several studies have shown that PDGF-β is overexpressed in glioma cell lines and its expression correlates with cellular proliferation [51, 52]. Seoane et al. showed that PDGF-β is involved in cellular proliferation induced by TGFβ in some glioma cell lines. The overexpression of PDGF-β is promoted by the increased TGFβ-Smad activity in glioma cell lines [53]. The molecular mechanistic study showed that TGFβ promotes glioma cell proliferation through the induction of PDGF-β in tumors with an unmethylated PDGF-β gene, which determines the TGFβ oncogenic response in glioma [53]. Harvouet et al have shown that folate supplementation limits the aggressiveness of glioma *in vitro* and *vivo* through the remethylation of some genes including PDGF-β [54, 55]. In our study, we demonstrate that TAZ promotes cell proliferation and colony formation in neuroblastoma cells through the induction of PDGF-β based on the following observations: (i) Overexpression of TAZ markedly increased the expression of PDGF-β in BE(2)-C cells, and enhanced PDGF-β protein was reduced back to their original levels when overexpressed TAZ in BE(2)-C cells was knocked down by TAZ siRNA; (ii) Knockdown of TAZ by siRNA markedly decreased the expression of PDGF-β in SK-N-AS cells, and decreased PDGF-β protein was restored back to their original levels when knocked down TAZ in SK-N-AS cells was overexpressed with TAZ; (iii) Knockdown of PDGF-β in TAZ overexpressing cells partially inhibited cell proliferation by causing cell cycle arrest at G1 phase and inhibiting the expression of cyclin D1. A clearer characterization of the functional role of TAZ in the activation of PDGF-β awaits further investigation.

In summary, the present study provided the important evidence that TAZ is an oncogene and has an important role in regulating cell proliferation and tumorigenesis in neuroblastoma cells. CTGF and PDGF-β are the major downstream transcriptional targets of TAZ. Our findings demonstrate that TAZ may act as a critical regulator of neuroblastoma cell proliferation and tumorigenicity, and seems to be a novel and promising target for the treatment of neuroblastoma.

## MATERIALS AND METHODS

### Cell culture and cell lines

The human neuroblastoma cell lines BE(2)-C [CRL-2268; American Type Culture Collection (ATCC)] was cultured in a 1:1 mixture of Dulbecco's modified Eagle's medium (DMEM) and Ham's nutrient mixture F12, SK-N-AS (CRL-2137; ATCC), SK-N-DZ (CRL-2149; ATCC), and SK-N-F1 (CRL-2141; ATCC) cells in DMEM. All culture medium were supplemented with 10% fetal bovine serum (Invitrogen), 1% benzylpenicillin and streptomycin. All cells cultured at 37°C in 5% CO_2_ humidified incubator.

### Lentiviral production, infection, and establishment of stable cell lines overexpressing or knocking down cellular genes

One day before transfection, 5 × 10^6^ 293FT cells were plated on 100-mm plate with lentiviral medium and incubated at 37°C overnight, Then 0.5 μg of pCDH-CMV-EF1-TAZ or pCDH-CMV-EF1-copGFP or pLKO.1 vector were mixed with 0.5 μg of pLP1, pLP2 and pLP/VSVG (packing) plasmids, and dilute 6 μL Lipofectamine™ 2000 with 250 μL OPTI medium (no serum, penicillin and streptomycin). After 20min at room temperature, the mixture was added dropwise into each 6 well plate, and add 850 μl of the 293FT cell suspension (1 × 10^6^ cells) to the plate containing medium and DNA-Lipofectamine™ 2000 complexes. Mix gently by rocking the plate back and forth, and incubate at 37°C in a humidified 5% CO_2_ incubator. The medium was replaced with lentiviral medium after 6–8 hours transfection. Two day after transfection, harvested virus-containing supernatants, pass through a 0.45-μm filter, and infected target cells, the second harvested virus after two days, when target cell growthing at 90% confluence, split to 100-mm plate with fresh target growth medium, the select cell with puromycin to gain the stable cell line.

### Immunofluorescent staining

Cells were grown on cover slips, washed with PBS, fixed in 4% paraformaldehyde (PFA) for 15min, and permeabilized with 0.3% Triton X-100 for 15min. Cells were blocked with 10% horse serum for 1h, incubated with a primary antibody for 2 hours and followed by incubation with the appropriate secondary antibody for 1 hour and 300 nM DAPI for counterstaining. Primary antibodies were 1:200, mouse anti-TAZ (560235; BD Biosciences), mouse anti-YAP (sc-101199; Santa Cruz Biotechnology), Alexa Fluor 488 goat anti-rat IgG (H + L) 1:500, and Alexa Fluor 594 goat anti-Mouse IgG (H + L) 1:500 (Invitrogen) were used as secondary antibodies. Nikon microscope with Image-Pro Plus software was used to examine and analyze the fluorescent signaling images.

### qRT-PCR

Cells were lysed with TRIzol (Takara) for total RNA purification. Reverse transcription was done by using Super-Script II Reverse Transcriptase (Invitrogen). qRT-PCR was done by a RT^2^ SYBR Green/Fluorescein PCR master mix (Takara). PCR reactions in triplicate were carried out by using an iQ5 real-time PCR systerm (Bio-Rad).

### Western blot assay

Proteins were extracted from neuroblastomas cells, and then separated by SDS-PAGE and transferred to a polyvinylidene difluoride membrane (PVDF). After blocked with 5% nonfat milk in TBST for 2 hours, the membrane was incubated with primary antibodies. Membranes were washed three times and incubated with the horseradish peroxidase-conjugated second antibodies. The signals were captured by the ECL reagent (Beyotime) and visualized by western blotting detection instruments (Clinx Science). Mouse anti-TAZ (560235; BD Biosciences), mouse anti-YAP (sc-101199, 1:200), goat anti-CTGF (sc-14939, 1:200) from Santa Cruz company, rabbit anti-PDGF-β (E1A0240-1, 1:1000) from EnoGene, and mouse anti-GAPDH (AG019, 1:1000) from Beyotime, cell cycle regulation antibody sampler kit #9932 from Cell Signaling Technology were used as primary antibodies. HRP-labeled goat anti-mouse IgG (H + L) (A0216, 1:5000) and goat anti-rabbit IgG (H + L) (A0208) were used as secondary antibodies which purchased from Beyotime.

### Cell counting kit-8 assay

Cell growth curve was determined by CCK-8 (Beyotime) assay. Cells were seeded onto 96-well plate at 1000 cells per well for overnight. 10 μL CCK8 was added to each well and incubated at 37°C for 2 hours, The absorbance was measured at a wavelength of 450 nm.

### Xenograft assay

Human neuroblastoma cell SK-N-AS transfect with GFPsi, TAZsi and TAZsi/TAZ respectively, grown in DMEM supplemented with 10% FBS. 4 weeks old NOD/SCID female mice (Beijing laboratory animal research center, China) were employed, both flanks were injected subcutaneously with 2 × 10^6^ cells in 200 μL of DMEM. After ten days, Tumor growth was estimated every two days by caliper measurements and tumor volume was calculated with the formula (volume = tumor length × width^2^ × 0.5236), Two weeks after tumor growth, tumors were removed, weighed, and paraffin-embedded. All animal studies were pre-approved by the Institutional Animal Care and Use Committees at Southwest University.

### Cell cycle assay

Cells were fixed in 70% ethanol, stained with propidium iodide (PI), and analyzed by flow cytometry (BD FACSCalibur system; BD BioSciences, San Jose, CA, USA). The data was analysed with CellQuest Pro (BD BioSciences).

### Patient data analysis

Patient data and gene expression datasets were obtained from the Oncogenomics Section Data Center (http://pob.abcc.ncifcrf.gov/cgi-bin/JK) and R2:microarray analysis and visualization platform (http://hgserver1.amc.nl/cgi-bin/r2/main.cgi). Kaplan-Meier analysis and resulting survival curves were done by using GraphPad Prism (version 6.0). All data and *P* values (log-rank test) were downloaded online, and all cutoff values for separating high and low expressing groups were determined by the online Oncogenomics algorithm [57].

### Statistical analysis

All experiments were set up in triplicates and the results were presented as mean ± S.D. Variance between the experimental groups was determined by 2-tailed Students *t* test, and a value of *P* < 0.05 was considered to be statistically significant.
